# Assessment of mechanical-loss property of 3D printing metal and its application to ultrasonic transducers as vibrating bodies^☆^

**DOI:** 10.1016/j.ultsonch.2025.107356

**Published:** 2025-04-15

**Authors:** Lipeng Wang, Ranxu Zhang, Jiang Wu, Chengqi Pan, Xiaoming Yue, Qiang Zhang, Yibin Li

**Affiliations:** a Center for Robotics, School of Control Science and Technology, Shandong University, Jinan 250061, China; b School of Mechanical Engineering, Shandong University, Jinan 250061, China; c National Key Laboratory of Underwater Acoustic Technology, Harbin Engineering University, Harbin 150001, China

**Keywords:** Ultrasonic transducer, Mechanical loss, Damping coefficient, 3D printing metal

## Abstract

As miniaturized ultrasonic transducers with sophisticated structure have become increasingly demanded, the vibrating bodies made by conventional metals face the problem of fabricating difficulty and high expenses. The 3D printing metals are prospective materials for their flexibility in forming complicated configurations, but their mechanical-loss properties need clarification as they greatly affect the vibration properties. As a pilot trail, first, an approach to measure the attention coefficients according to the distributions of the vibration velocity and the phase was developed to evaluate their dependence on the strain and the frequency. Then, an aluminum alloy via 3D printing (AlSi10Mg) was employed as the vibrating bodies to form the ultrasonic transducers, whose performance, e.g., vibration properties, temperature rise, and sound pressure level (SPL) in water, was assessed and compared with conventional aluminum alloy (7075). As typical results, AlSi10Mg’s damping coefficient is 1.16 times that of 7075 at 33 kHz frequency; this implies the 3D printing process does not deteriorate the aluminum alloy’s mechanical-loss property. Meanwhile, AlSi10Mg’s damping coefficient reaches 2.19 × 10^-4^ at the laser power of 350 W, relatively small compared to the values corresponding to other laser powers; this indicates the capability to reduce the mechanical loss by adjusting the parameters during 3D printing possesses. Moreover, the maximum vibration velocity and the SPL of the AlSi10Mg transducer are 1.13 and 1.11 times those of the 7075 transducer that has the same configuration and operates in the same vibration modes. This study enriches the candidate materials as the vibrating bodies of ultrasonic transducers, which potentially meet the demands in wider ultrasonic application fields.

## Highlights

• An approach to evaluate the damping coefficient’s dependence on the strain and the frequency was developed based on the acoustic intensity theory.
• AlSi10Mg’s damping coefficient is 1.16 times that of 7075 at 33 kHz frequency; this implies the 3D printing process does not deteriorate the aluminum alloy’s mechanical-loss property.
• AlSi10Mg’s damping coefficient reaches 2.19 × 10–4 at the laser power of 350 W, relatively small compared to those at the other laser powers, because the numbers and the dimensions cavities are relatively small.
• The maximum vibration velocity and the SPL of the AlSi10Mg transducer are 1.13 and 1.11 times those of the 7075 one with the same structure and the same vibration mode.

## Graphical abstract

**Figure:**
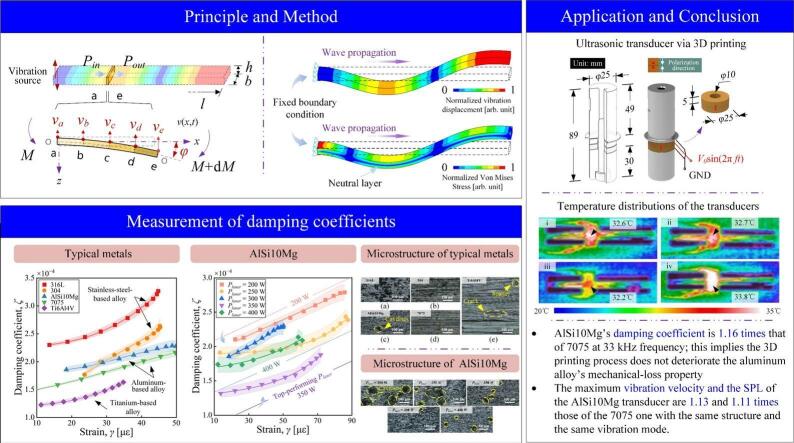

## 1 Introduction

As the key components of ultrasonic devices, ultrasonic transducers have been applied to various fields, e.g., synthesis of new materials, ultrasound-assistant chemical reactions, and acoustic cavitation [[1], [2], [3]]. Composed by piezoelectric ceramic elements and vibrating bodies, the ultrasonic transducers convert electrical energy into vibrational energy and generate the vibrations in various modes [4,5]. Since the transducers should provide sufficient vibration intensity, it is essential to reduce the energy loss [6,7]. In general, the energy loss includes the dielectric and mechanical ones. The dielectric loss originates from crystal dislocations or vacancies inside piezoelectric ceramic elements as well as the polarization hysteresis, and its reduction is usually achieved by optimizing the recipe of piezoelectric ceramic elements [8]. Since the ultrasonic transducers commonly work in several tens of kilohertz or several megahertz, many factors, e.g., invisible cavity (resulting in scattering [9,10]) or internal friction on the layers of crystal grains (extensively existing in the high-frequency region [11]) lead to the mechanical loss when the ultrasounds propagate through the vibrating bodies, which takes far larger parts in energy loss than dielectric loss. Thus, by basically considering the mechanical loss (as well as other properties), selecting suitable material(s) as the vibrating bodies is a fundamental step prior to designing/fabricating the ultrasonic transducers.

Engineering materials dominantly include metals, fine ceramics, and polymers, and their mechanical-loss properties have been explored in previous literatures. Here, the mechanical-loss property is commonly indicated as the damping coefficient. Concerning the metals, Karafi et al. [12] developed a Langevin transducer composed by aluminum 7075-T6 and stainless steel 304, and found that the mechanical loss in the vibrating bodies takes 83.1 % of energy loss. Nader et al. [13] designed an aluminum transducer operating in the flexural vibration and reported that its damping coefficient was 7.2 × 10^-3^ at 48 kHz frequency. Karafi and Kamali [14,15] investigated the damping characteristics of steel and titanium, and revealed that the damping coefficient of the titanium bar (0.96 × 10^-3^) was approximately 4.6 times higher than that of the steel bar (0.21 × 10^-3^). Sciegaj et al. [16] studied the ultrasonic attenuation in steel bars and found that the damping behavior is significantly affected by the stress state. In terms of practical usage of ultrasonic transducers, metals usually possess moderate damping coefficients (compared to fine ceramics and polymers), which make them applicable to the transducers with regular structure and general dimensions. However, with the development of miniature ultrasonic actuators and small airborne ultrasonic transmitters [7,17], irregular configurations become increasingly demanded to enhance the vibration intensity of transducers [6,7,18], but they unavoidably increase the difficulty and/or the expense during the complicated processes of making the metal components. Regarding the fine ceramics, since they belong to atomic crystal, their regular atomic (or molecular) frameworks facilitate the suppression of scattering [9,19], which allows fine ceramics to have relatively low mechanical loss. For instance, Wolfenden et al. [20] reported that alumina’s damping coefficient was 0.05 × 10^-3^. However, the fine ceramics’ fragility as well as high fabricating expenses restrict their practical application in transducers. In terms of polymers, owing to the non– or semi-crystalline frameworks, their damping coefficients are reported to be > 20 × 10^-3^, making most polymers suitable for suppressing the vibration [21,22]. As a special case, Wu et al. [23] found that the damping coefficient of a semi-crystalline polymer, namely poly phenylene sulfide (PPS), was ∼ 0.1 times the values of most polymers and comparable to those of some metals [24,25]. Though PPS’s low mechanical loss and low density can enhance the vibration intensity, its low elastic modulus causes the mismatching between the PPS vibrating body and the piezoelectric ceramic elements, which limits the power density when the PPS-based transducers are utilized as the actuators [[21], [22], [23], [24], [25]]. Considering the obstacles of conventional engineering materials, it would be meaningful to test newly-developed material(s) capable of balancing the mechanical/acoustic properties and the fabricating ease/expense to develop ultrasonic transducers.

Recently, metal three-dimensional (3D) printing has become an attractive technology as it endows the rapid prototyping of the structure with micro sizes and/or complicated shapes [26,27], which infers that the vibrating bodies made by 3D printing metal are the candidate materials for these reasons: First, the capability to make the micro-sized structure guarantees the fabrication of small transducers (e.g., the tiny transducer array for sending out airborne ultrasounds with changeable directivity [26,27]) or part of the Langevin transducer that uses complicated structure to suppress the mechanical loss (e.g., small slots near the contacting surfaces that are in contact with the piezoelectric ceramic elements [28]). On the other hand, the components with 3D printing metal can decrease the expense compared to those with conventional metals as the cutting tools specially designed for irregular structure are not needed. Second, the adjustable parameters in 3D printing, e.g., laser power, scanning speed, or layer thickness, make it possible to actively change the mechanical/acoustic properties. For example, Bakhtiarian et al. [29] revealed that the density and the hardness of stainless steel 316L were affected by the laser power and the scanning speed. Liverani et al. [18] reported that, by changing the laser power during the 3D printing of stainless steel 316L, there existed the cavities whose sizes could be controlled to a certain extent. As an instructive study, Wang et al. [30] designed a longitudinal/torsional transducer whose twisting-shaped vibrating body was fabricated through the selective-laser-melting 3D printing process. These reports imply that it is feasible to utilize the 3D printing metals as the vibrating bodies of ultrasonic transducers, but the following problems still exist.

(1) The approach to measure the damping coefficient of 3D printing metal is absent. For the impact testing method [12,16] and the resonance curve method [[13], [14], [15]], the results reflect the mechanical loss of the entire structure apart from the vibrating body; and the traveling-wave-based method [31,32] only reflects the damping coefficient in the low-amplitude region. Besides, the dependence of mechanical loss on the strain and the frequency, necessary for designing the transducers and deciding the optimal actuating parameters, needs quantitative analysis.

(2) The relationship between the damping coefficient and the parameters in fabricating the 3D printing metal needs clarification; this would be an initial step to state the technical possibility to reduce the mechanical loss by adjusting the parameters during the process of 3D printing.

To address these problems, we investigate the mechanical-loss properties of AlSi10Mg (a typical 3D printing metal) and tested its application to the ultrasonic transducers. Initially, on the basis of acoustic intensity theory [31,32], an approach to measure the damping coefficient according to the vibration velocity and the phase was developed to evaluate how the damping coefficient depends on the frequency and the strain; this work can be regarded as the contribution of this article. Subsequently, a series of experiments and the comparison with conventional metals were carried out to show the mechanical-loss properties of AlSi10Mg. In specific,

(1) Within the strain range of 18 to 50 με (corresponding to the vibration velocities from 53.80 to 149.44 mm/s), the damping coefficient of AlSi10Mg increases from 1.8 × 10^-4^ to 2.3 × 10^-4^. As the frequency approaches 45 kHz, the damping coefficient reaches 6.1 × 10^-4^, close to the values of aluminum but smaller than those of stainless steel.

(2) The damping coefficient of AlSi10Mg fabricated at the laser power of 350 W is smaller than those corresponding to other laser powers since the numbers and/or the size of cavities are smaller.

(3) When the vibrating bodies made of AlSi10Mg are assembled in the transducer, its maximum vibration velocity and maximum sound pressure level are respectively 12.76 % and 11.30 %, larger than the values of the 7075 transducer that has similar structure and operates at almost the same frequency.

The rest of this article are organized as follows. Section II describes the method to measure the damping coefficient. Section III shows the experimental results of the damping coefficients of AlSi10Mg with varying laser power. Section IV compares the vibration properties of the transducers made of AlSi10Mg and 7075. Section V concludes this article.

## 2 Principle and method

### 2.1 Principle

**Fig. 1:**
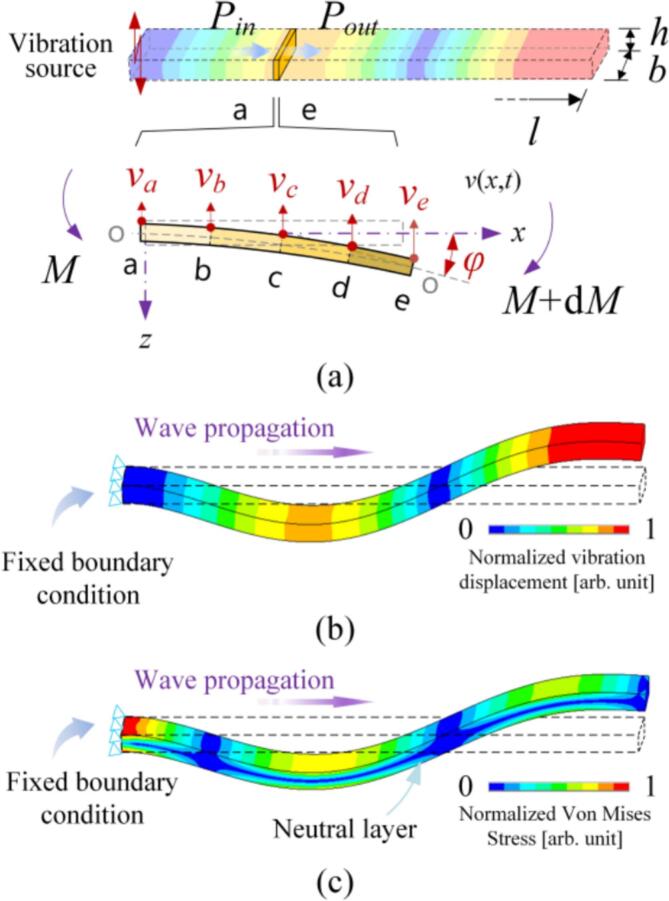
Conceptual view of measuring the damping coefficient. (a) Calculation of the reactive energy and the active energy from the vibration velocities and the phases that are measured at adjacent five points a–e. (b) The vibration displacement distribution. (c) The stress distribution.

As shown in Fig. 1(a), the vibration source is fixed at one end of a rectangular beam, whose length, width, and thickness are indicated as *l*, *b*, and *h*, respectively. During the excitation of a flexural wave, the energy flows through a micro element. Here, the energy flowing into the element and out of the element are indicated as *P_in_* and *P_out_*, respectively. According to the acoustic intensity theory [31,32], the damping coefficient is defined as the ratio of the dissipated energy (power) *E_p_* (*P_p_*) to the reactive energy (power) *E_k_* (*P_k_*) within one period. In specific,

(1) $$\zeta =\frac{E_{p}}{4\pi E_{k}}=\frac{E_{p}/\left(2\pi f\right)}{4\pi E_{k}/\left(2\pi f\right)}=\frac{1}{4\pi }\cdot \frac{P_{p}}{P_{k}},$$

where *f* means the frequency. In this study, *P_p_* is the reduction in active power flowing through the cross-sections b and d, while *P_k_* as the reactive power stored in the sub-elements between b and d. Accordingly, Eq. (1) can be rewritten as

(2) $$\zeta =\frac{1}{4\pi }\cdot \frac{P_{p-d}-P_{p-b}}{P_{k(b∼c)}+P_{k(c∼d)}}.$$

In the flexural wave, the moment *M* applied to the micro element causes the deflection angle *φ*. In this case, *P_k_* and *P_p_* can be expressed as [31,32]

(3) $$\left\{\begin{array}{c} P_{k}=\text{Im}\left(M\cdot \varphi *\right)\\ P_{P}=\text{Re}\left(M\cdot \varphi *\right) \end{array}\right),$$

According to the Bernoulli-Euler model [31,32], *M* and *φ* can be derived from the vibration velocity *v* with the following equations [32]:

(4) $$M=\frac{EI_{z}}{j\cdot 2\pi f}\cdot \frac{\partial^{2}v}{\partial x^{2}}$$

and

(5) $$\varphi =\frac{1}{2\pi f}\cdot \frac{\partial v}{\partial x},$$

respectively, where *j* means the imaginary unit, *E* denotes the elastic modulus, and *I_z_* (=*bh*^3^/12) is the moment of inertia. Substituting Eqs. (4), (5) into Eq. (3), we obtain that

(6) $$P_{k}=\text{Im}\left(M\cdot \varphi^{*}\right)=\text{Im}\left[\frac{EI_{z}}{j\cdot 2\pi f}\cdot \frac{d^{2}v}{dx^{2}}{\left(\frac{\mathrm{dv}}{\mathrm{dx}}\right)}^{*}\right]$$

and

(7) $$P_{p}=\text{Re}\left(M\cdot \varphi^{*}\right)=\text{Re}\left[\frac{EI_{z}}{j\cdot 2\pi f}\cdot \frac{d^{2}v}{dx^{2}}{\left(\frac{\mathrm{dv}}{\mathrm{dx}}\right)}^{*}\right].$$

Supposing that the velocity velocities at the sub-sections a-e are expressed as

(8) $$\left\{\begin{array}{c} v_{a}=V_{a}{\text{e}}^{j(2\pi ft+\theta_{a})}\\ v_{b}=V_{b}{\text{e}}^{j(2\pi f+\theta_{b})}\\ v_{c}=V_{c}{\text{e}}^{j(2\pi f+\theta_{c})}\\ v_{d}=V_{d}{\text{e}}^{j(2\pi f+\theta_{d})}\\ v_{e}=V_{e}{\text{e}}^{j(2\pi f+\theta_{e})} \end{array}\right),$$

where *V_a_*, *V_b_*, *V_c_*, *V_d_*, and *V_e_* mean the amplitudes while *θ_a_*, *θ_b_*, *θ_c_*, *θ_d_*, and *θ_e_* denote the phases. By substituting Eq. (10) into Eq. (9), we obtain the following equations:

(9) $$\begin{array}{c} P_{k-bd}=P{\;}_{k(b∼c)}+P_{k(c∼d)}\\ =\frac{EI_{z}}{4\pi f}\cdot \frac{1}{{\left(\Delta x\right)}^{3}}\left[V_{b}^{2}-V_{d}^{2}-2V_{b}V_{c}\cos\left(\theta_{b}-\theta_{c}\right)+2V_{c}V_{d}\cos\left(\theta_{c}-\theta_{d}\right)\right], \end{array}$$

(10) $$P_{p-b}=\frac{EI_{z}}{4\pi f}\cdot \frac{1}{{\left(\Delta x\right)}^{3}}\left[2V_{a}V_{c}\sin\left(\theta_{c}-\theta_{a}\right)+2V_{b}V_{c}\sin\left(\theta_{b}-\theta_{c}\right)+2V_{a}V_{b}\sin\left(\theta_{a}-\theta_{b}\right)\right],$$

and

(11) $$P_{p-d}=\frac{EI_{z}}{4\pi f}\cdot \frac{1}{{\left(\Delta x\right)}^{3}}\left[2V_{c}V_{e}\sin\left(\theta_{e}-\theta_{c}\right)+2V_{d}V_{e}\sin\left(\theta_{d}-\theta_{e}\right)+2V_{c}V_{d}\sin\left(\theta_{c}-\theta_{d}\right)\right],$$

where Δ*x* means the distance between the adjacent sampling points. The above equations ensure the derivation of *ζ* at different positions of a beam. Meanwhile, as shown in Fig. 1(b) and (c), the stress (or strain) differs at different positions; this allows us to obtain how *ζ* depends on the strain. Moreover, with shifting *f*, the stress (or strain) at one certain position changes as a consequence of the variation of the nodes; this provides the way to measure the dependence of *ζ* on *f* [16,32].

### 2.2 Method

(1) *Deciding the mechanical parameters:* The 3D-printing metals including 316L (the stainless-steel-based alloy), AlSi10Mg (the aluminum-based alloy), and Ti6Al4V (the titanium-based alloy) were prepared and their properties were compared with conventional metals, namely 304 (stainless steel) and 7075 (aluminum). Their mechanical constants, e.g., *E*, Poisson's ratio *μ*, and density *ρ* are listed in Table 1. Though *E*, *μ*, and *ρ* are different among these materials, their longitudinal wave speeds (indicated as the square root of *E*/*ρ*) do not markedly differ.

**Table 1:** Mechanical constants of the tested materials.

	Material	Elastic modulus, *E* (GPa)	Poisson's ratio, *μ*	Density, *ρ* (×10^3^ kg/m^3^)	$\sqrt{E/\rho }$(×10^3^ m/s)
3D Printing metal	316L	164	0.30	7.11	4.80
	AlSi10Mg (Laser power: 300 W)	71	0.33	2.65	5.18
	Ti6Al4V	127	0.33	4.47	5.33
Conventional metal	304	197	0.29	8.14	4.92
	7075	70.3	0.31	2.87	4.95

**Fig. 2:**
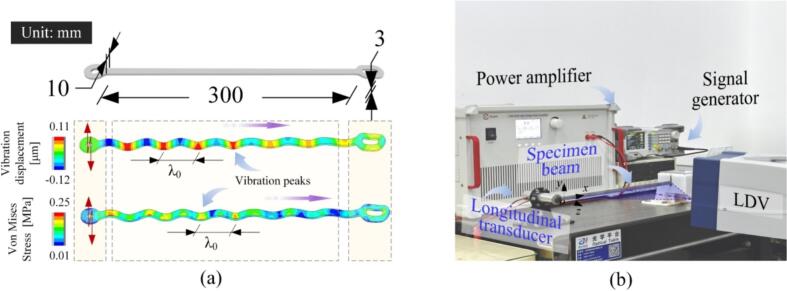
(a) Key dimensions of the beam. (b) The experimental setup for measuring the distributions of the vibration velocity and the phase. Here, LDV means the laser Doppler vibrometer.

(2) *Constructing the experimental setup:* Fig. 2(a) illustrates the beam working in the flexural vibration. As shown in Fig. 2(b), some longitudinal transducers act as the vibration sources to send out the ultrasounds in the frequency range of 20–50 kHz. A sinusoidal signal was provided with a signal generator (DG1022Z, RIGOL, Beijing, China) and amplified with a bipolar power amplifier (ATA-4052, Aigtek Techno., Xi’an, China). The *x*-axis distributions of the vibration velocities and the phases were measured with a scanning laser Doppler vibrometer (PSV-500, Polytec, Waldbronn, Germany).

(3) *Setting* Δ*x and h:* To determine these parameters, the flexural wavelength is derived as [32]

(12) $$\lambda^{2}=\frac{2\pi }{f}\cdot \sqrt{\frac{EI_{z}}{\rho A}}=1.81\cdot \frac{h}{f}\cdot \sqrt{\frac{E}{\rho }}.$$

Here, *f* is, as mentioned above, in the range from 20 to 50 kHz. According to the sampling theorem in the spatial domain [31,32], it is recommended that Δ*x* equals *λ*/20. When *h*s are 1, 3, and 5 mm, *λ*s are in the ranges of 13–21, 23–36, and 31–46 mm, corresponding to Δ*x* = 0.65, 1.15, and 1.55 mm, respectively. Since the beam with a large *l*/*h* ratio easily causes the redundant vibration (e.g., the twisting vibration) [7,35,36], it is desirable that the beam has a relatively large *h*. On the other hand, a relatively small value of *h* facilitates the enhancement in *λ*, which makes the decayed waveform observable [16]. In a balancing aspect, we set *h* to 3 mm, and accordingly, Δ*x* = 1.15 mm, *l* = 300 mm, and *b* = 10 mm.

## 3 Measurement of damping coefficients

### 3.1 Case study

**Fig. 3:**
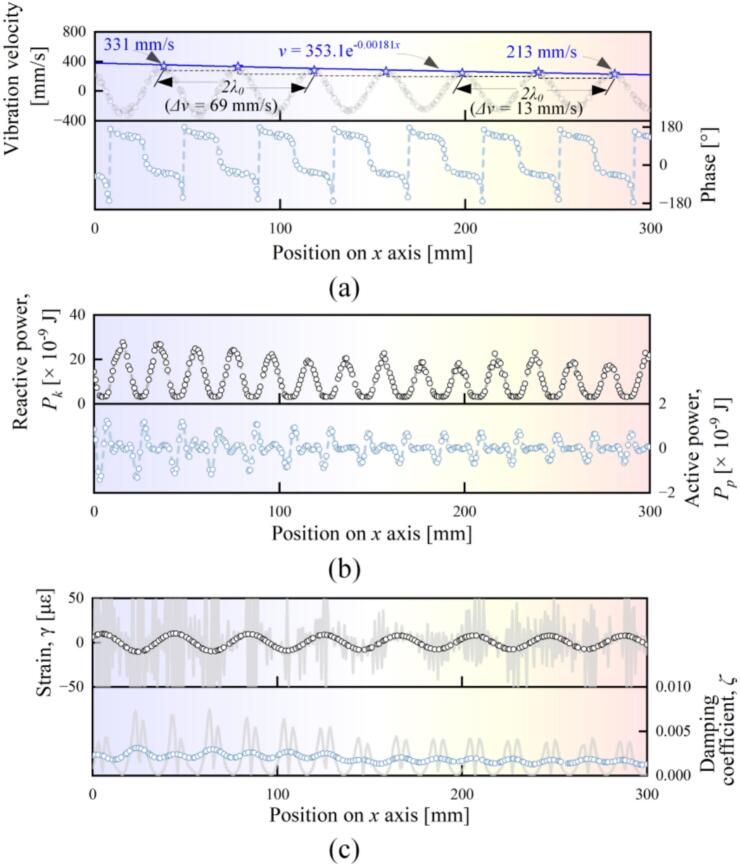
An example of calculating the damping coefficient. (a) The vibration velocity and the phase of a flexural beam. (b) The distribution of the reactive energy and the active energy. (c) The distribution of the strain and the damping coefficient. Here, the original data is given as the gray curves, and the results processed by the wavelet analysis are given as the dots.

Prior to investigating the mechanical-loss properties of various materials, an example of measuring *ζ* was given to illustrate the procedures. Fig. 3(a) shows the *x*-axis distributions of the vibration velocity and the phase on an AlSi10Mg beam when *f* and the driving voltage were set to 23.8 kHz and 50 V, respectively. Clearly, more than seven wavelengths with the decayed tendency existed as the wave propagated along the + *x* axis. Besides, the vibration velocity provided the peak value of 331 mm/s at *x*  = 35 mm. Fig. 3(b) shows how *P_k_* and *P_b_* are distributed on the beam. The maximum value of *P_k_* appears when the vibration velocity reaches the positively or negatively maximum value (which means more energy is stored at the positions where more intensive vibration is excited), while the maximum value of *P_b_* (or the intensive mechanical-loss) exists at the nodes (where the vibration velocity approaches zero). Besides, both *P_k_* and *P_p_* exhibited the tendencies of reduction as the wave propagated along the + *x* axis. Fig. 3(c) illustrates how *γ* (the strain) and *ζ* are distributed on the beam. Here, *γ* is derived with the following equation [31,32]:

(13) $$\gamma =\frac{h}{4\pi f}\cdot \frac{\partial^{2}v}{\partial x^{2}}.$$

The wavelet analysis was applied to the original data to suppress the noise in the high frequency range [34], where the basis function of wavelet was ‘db4′ and the decomposition level was set to 4. As typical values, in the *x* range of 20–167 mm, *ζ* decreased from 3.01 × 10^-3^ to 1.33 × 10^-3^ as *γ* decreased from 20 to 17 με. The mean value of *ζ* was 1.94 × 10^-3^ with the standard deviation of 1.72 × 10^-3^.

### 3.2 Damping coefficients among the tested materials

**Fig. 4:**
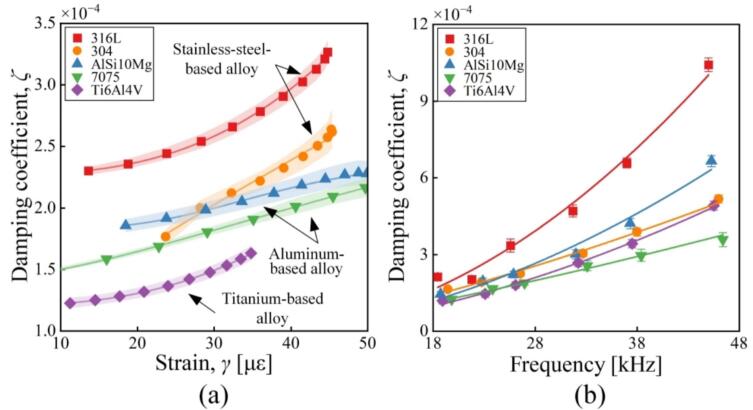
Damping coefficients of typical materials as functions of (a) the strain and (b) the frequency.

First, *ζ*s of the tested materials were explored with varying *γ* and *f*. As shown in Fig. 4(a), *ζ* shows the increasing tendency as *γ* become larger. For the stainless steel and aluminum alloys, *ζ*s are slightly higher for the 3D printing metals than for the conventional ones. Among the 3D printing metals, *ζ*s of 316L are relatively high compared to AlSi10Mg and Ti6Al4V. The minimum values of *ζ* are 1.94 × 10^-3^ and 1.27 × 10^-3^ when *γ*s of AlSi10Mg and Ti6AlV are 18.5 με and 11.2 με, respectively. Fig. 4(b) shows the relationship between *ζ* and *f*. It is observable that there exists the enhancement in *ζ* with increasing *f*. The frequency range of 18–38 kHz is heavily adopted for high-power ultrasonic usage, e.g., airborne ultrasounds (28 or 35 kHz) [37,38] and ultrasonic welding (20 kHz) [39], and the ζs of AlSi10Mg in this range approach the values of conventional metals; this allows us to predict that the substitution with AlSi10Mg does not greatly affect the transducers’ vibration properties as well as the ultrasonic usage. In the range of 38–48 kHz, the ζs of 316L exceed those of conventional metals; this should originate from the cavities, which is one type of internal defects [9]. Whereas the ζs of AlSi10Mg do not greatly differ from those of conventional metals.

**Fig. 5:**
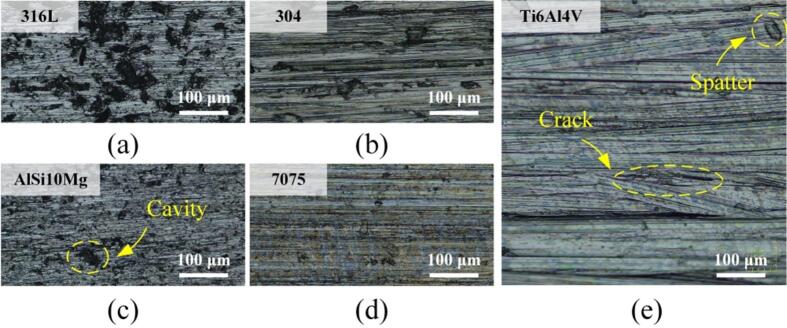
Morphologies of (a) 316L, (b) 304, (c) AlSi10Mg, (d) 7075, and (e) Ti6Al4V.

To verify the tendency given in Fig. 4, the morphologies of these materials were investigated with an optical microscope (DSX1000, Olympus, Tokyo, Japan). As shown in Fig. 5(a) and (b), more cavities are observable on the surface of 316L than on that of 304; this may explain why *ζ*s exceed those of 304 and the other tested materials regardless of *γ* or *f*. The numbers and the dimensions of cavities are larger for AlSi10Mg’s surface [see Fig. 5(c)] than for that of 7075 [see Fig. 5(d)]. As shown in Fig. 5(e), Ti6Al4V’s surface is relatively smooth compared to those of the others, though tiny cracks or spatters can be observed here.

### 3.3 Damping coefficient of AlSi10Mg versus laser power

**Fig. 6:**
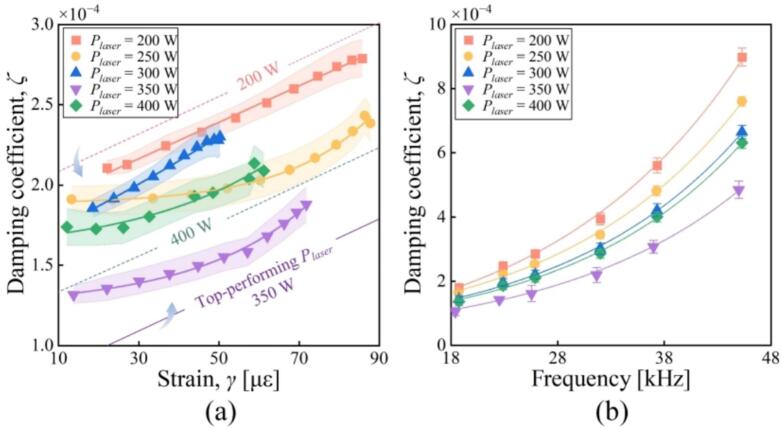
Damping coefficient of AlSi10Mg as functions of (a) the strain and (b) the frequency when various laser powers are applied during the manufacturing of AlSi10Mg.

**Fig. 7:**
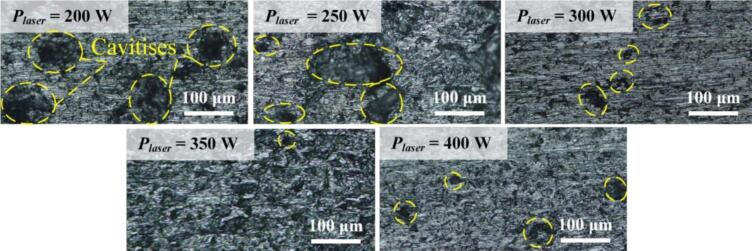
Morphologies of AlSi10Mg at the laser powers of 200, 250, 300, 350, and 400 W.

Then, *ζ*s of AlSi10Mg were explored as functions of the laser power, while the other parameters are listed in Table 2. As shown in Fig. 6(a), *ζ* corresponding to the laser power of 350 W decreased to 34.1 % of that to the laser power of 200 W. However, when the laser power increased to 400 W, unexpectedly, *ζ* increased to ∼ 2 times the value corresponding to 350 W. As shown in Fig. 6(b), the *ζ*-to-*γ* relationships show identical tendencies though varying laser powers are varied. Meanwhile, *ζ*s obtained at 350 W laser power are relatively low compared to those at the other laser powers. These tendencies may be explained with the morphologies of AlSi10Mg fabricated at varying laser power [see Fig. 7], where the dimensions and the numbers of cavities decrease as the laser power ranges from 200 to 350 W but more cavities appear at 400 W. As a typical result, *ζ* of AlSi10Mg fabricated at 350 W laser power was 4.85 × 10^-3^, equal to 1.34 times that of 7075; this implies that AlSi10Mg can serve as an alternative vibrating body in ultrasonic transducers.

**Table 2:** Parameter settings for fabricating AlSi10Mg specimens.

Parameters	Laser power, *P_laser_* (W)	Scanning speed (mm/s)	Laser spot diameter (μm)	Layer thickness (μm)	Spacing of profiles lines (μm)
value	200–400	1200	80	30	100

## 4 Ultrasonic transducer via 3D printing

### 4.1 Structure

**Fig. 8:**
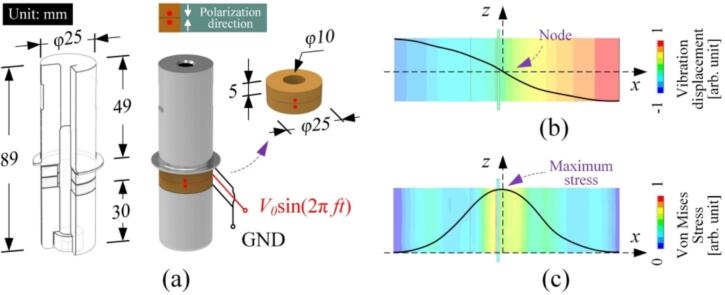
Langevin transducer tested in this study. (a) The configuration, (b) the vibration displacement distribution, and (c) the stress distribution.

The Langevin transducers were constructed to test the feasibility of fabricating the ultrasonic transducers via 3D printing. It is worth mentioning that AlSi10Mg is chosen for its relatively small damping coefficient compared to 316L as well as its relatively low expense compared to Ti6Al4V (for the specimens with almost the same volumes, AlSi10Mg’s expense is 0.28 times that of Ti6Al4V). Fig. 8(a) illustrates the configuration, where the length and diameter are 89 and 25 mm, respectively. Each transducer is composed of two lead-zirconate-titanite (PZT) disks 25 mm in diameter and 5 mm in thickness. As shown in Fig. 8(b), the transducer works in the 1st longitudinal vibration. The maximum stress exists at the node, where the PZT disks are in contact with the AlSi10Mg vibrating bodies.

### 4.2 Finite element analysis

**Fig. 9:**
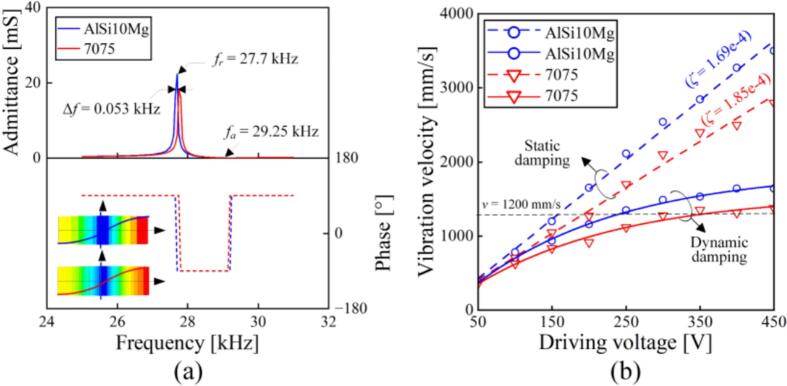
Finite element analysis of transducers made by AlSi10Mg and 7075. (a) Admittance characteristics. (b) The vibration velocity as functions of the driving voltage.

The finite element analysis (FEA) was performed to evaluate the acoustic performance of the transducers made by AlSi10Mg and 7075. Fig. 9(a) shows the admittance curves of the transducers, where only longitudinal vibrations are excited. Fig. 9(b) illustrates how the vibration velocities at the ends of the transducers vary as the driving voltages change. When the static damping was set into the simulation, the vibration velocities exhibited an approximately linear enhancement as the driving voltages become larger. However, when the driving voltage increases, the damping became higher because of more intense energy dissipation [9,31,32]. As a result, dynamic damping, whose damping parameters are given in Fig. 4, Fig. 6, was set in the simulation. Clearly, the vibration velocities of both AlSi10Mg and 7075 transducers show the saturation in the high-amplitude region.

### 4.3 Vibration properties

#### 4.3.1 Admittance characteristics

**Fig. 10:**
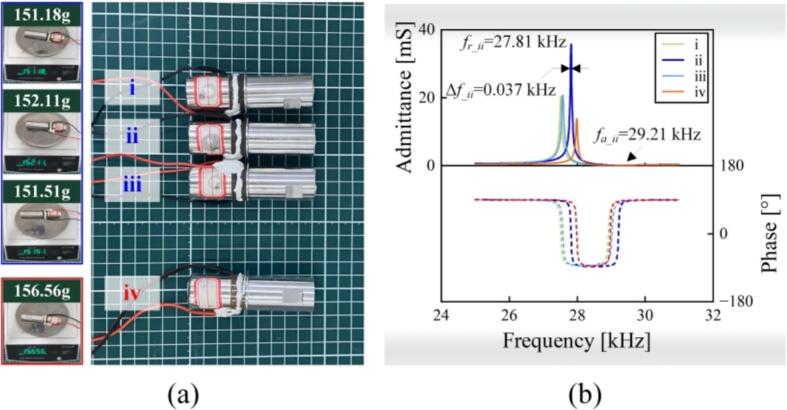
(a) Prototypes and (b) admittance characteristics of the transducers made by AlSi10Mg (transducers i, ii, and iii), 7075 (transducer iv).

Initially, the vibration properties of the transducers via 3D printing were explored. As shown in Fig. 10(a), the vibration bodies of the transducers labeled as i, ii, and iii were made of AlSi10Mg fabricated at the laser power of 350 W, while that of the transducer, labeled as iv, was made of 7075. Fig. 10(b) shows the admittance curves, where the resonant frequency *f_r_*, anti-resonant frequency *f_a_*, motional admittance *Y_m0_*, mechanical quality factor *Q_m_*, and clamped capacitance *C_d_* are directly obtained. The electromechanical coupling factor *k*, motional capacitance *C_m_*, motional resistance *R_m_*, and motional induction *L_m_* are derived with the following equations:

(14) $$k=\sqrt{1-{\left(\frac{f_{r}}{f_{a}}\right)}^{2}},$$

(15) $$C_{m}=\frac{Y_{m0}}{2\pi f_{r}Q_{m}},$$

(16) $$R_{m}=\frac{1}{Y_{\mathrm{mo}}},$$

and

(17) $$L_{m}=\frac{Q_{m}}{2\pi f_{r}Y_{m0}},$$

respectively. Table 3 lists the results. It could be found that *f_r_*s of the AlSi10Mg transducers ranged from 27.53 to 27.81 kHz, slightly lower than that of the 7075 one (27.99 kHz). The AlSi10Mg transducer ii’s *Y_m0_* reached 35.7 mS, ∼2.56 times that of the 7075 one. As a consequence, the AlSi10Mg transducer iii exhibited stronger electromechanical coupling (*k* = 30.69 %) and smaller mechanical loss (*Q_m_* = 737) than the 7075 transducer (*k* = 25.66 % and *Q_m_* = 538).

**Table 3:** Equivalent circuit parameter of the tested transducers.

	AlSi10Mg	AlSi10Mg	AlSi10Mg	7075
Label	i	ii	iii	iv
Resonant frequency *f_r_* [kHz]	27.53	27.81	27.57	27.99
Anit-resonant frequency *f_a_* [kHz]	28.92	29.21	29.03	28.96
Motional admittance *Y_m0_* [mS]	20.4	35.7	20.4	13.9
Mechanical quality factor *Q_m_*	426	737	470	538
Clamped capacitance *C_d_* [nF]	2.14	2.14	2.14	2.14
Electromechanical coupling coefficient *k* [%]	30.63 %	30.69 %	31.31 %	25.66 %
Motional capacitance *C_m_* [nF]	0.276	0.277	0.250	0.147
Motional resistance *R_m_* [Ω]	49.0	28.0	49.0	71.9
Motional inductance *L_m_* [H]	0.12	0.12	0.13	0.22

#### 4.3.2 Vibration velocity

**Fig. 11:**
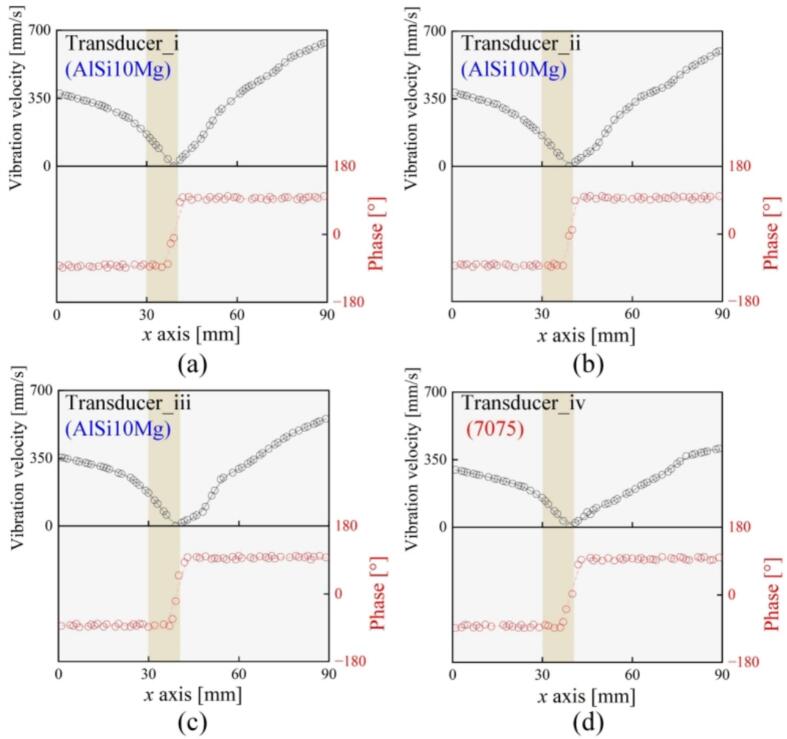
Vibration velocity distributions of the transducers made by (a) AlSi10Mg-i, (b) AlSi10Mg-ii, (c) AlSi10Mg-iii, and (d) 7075-iv.

Subsequently, the vibration velocity distribution of each transducer was measured. Here, the in-plane vibration velocity was measured with a laser Doppler vibrometer (IPV100, Polytec, Waldbronn, Germany). The results of the AlSi10Mg transducers i, ii, and iii are given in Fig. 11(a), (b), and (c), respectively, while that of 7075 is given in Fig. 11(d). At the driving voltage of 50 V, the maximum vibration velocity of the AlSi10Mg transducer i reached 633.3 mm/s, ∼1.56 times that of the 7075 transducers. Thus, the relatively high vibration velocity as well as the small deviation in *f_r_*s imply the AlSi10Mg transducer’s vibration property is not inferior compared to the 7075 one.

**Fig. 12:**
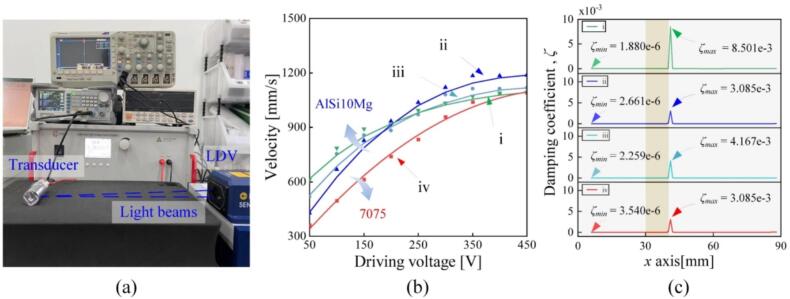
Vibration properties. (a) Experimental setup. (b) The vibration velocity as functions of the driving voltage. (c) The damping coefficients at different positions.

As additional assessment, the vibration velocities at the transducers’ free ends were measured when the driving voltages were varied [see Fig. 12(a)]. As shown in Fig. 12(b), the vibration velocity initially increased in a nearly linear way as the driving voltage became larger, while it leveled off when the driving voltages were 350 V for the AlSi10Mg transducers, and 400 V for the 7075 transducer. Besides, the maximum vibration velocity of the AlSi10Mg transducer ii reached 1184 mm/s, while the value of the 7075 aluminum was 1050 mm/s; this should be caused by the saturation in PZT’s vibration in the high-amplitude region [31] as well as the discrepancy in the preloads during the transducer’s assembly [32]. Fig. 12(c) shows the damping coefficients at different positions of the transducers, calculated by the method developed in this study. For each transducer, the damping coefficients reached the maximum and minimum values at the node and the free end, respectively; while their ratio is three orders of magnitude. The minimum damping coefficient at the AlSi10Mg transducer ii is 0.75 times that of the 7075 one (iv), while the maximum damping coefficients are close for these two transducers. Besides, the damping coefficients of the transducers (in contrast to those of the materials) are influenced by not only the ultrasonic attenuation inside the vibrating bodies but also the contact status between the vibration bodies and the PZT disks (which is not easy to keep uniform as a consequence of assembly); this can explain why the damping coefficients differ among the AlSi10Mg transducers.

### 4.4 Temperature rise

**Fig. 13:**
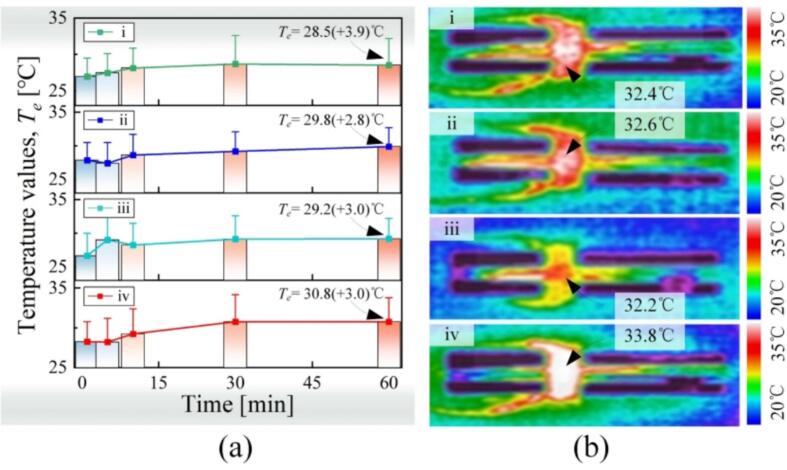
Temperatures on the transducers’ surfaces. (a) The variations in the averaged temperatures under continuous excitation. (b) The temperature distributions.

Followingly, the temperature of the transducers’ surfaces *T_e_* were monitored with an infrared camera (UTi120T, Uni-Trend Techno., Guangzhou, China) during the continuous excitation of ∼ 60 min. The initial *T_e_* was the environment temperature (23.8 °C). Fig. 13(a) illustrates the mean *T_e_*s in the time domain. The mean *T_e_* showed gradual enhancement and saturated at ∼ 30 min. At 60 min, the maximum *T_e_*s of the AlSi10Mg transducers were in the range of 28.5 °C–29.8 °C, smaller than that of the 7075 transducer (30.8 °C). However, at 60 min, the *T_e_*’s deviations of the AlSi10Mg transducers i and iii are not smaller than that of the 7075 transducer because of more energy dissipation near the node, which can be illustrated with the thermal images given in Fig. 13(b).

### 4.5 Sound pressure in water

**Fig. 14:**
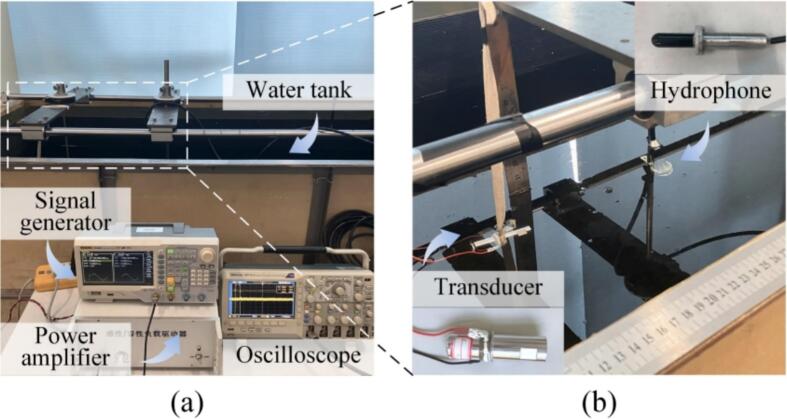
Measurement of the sound pressure level provided with the transducers. (a) The entire testbed. (b) The arrangement of the transducer and the hydrophone inside the water tank.

**Fig. 15:**
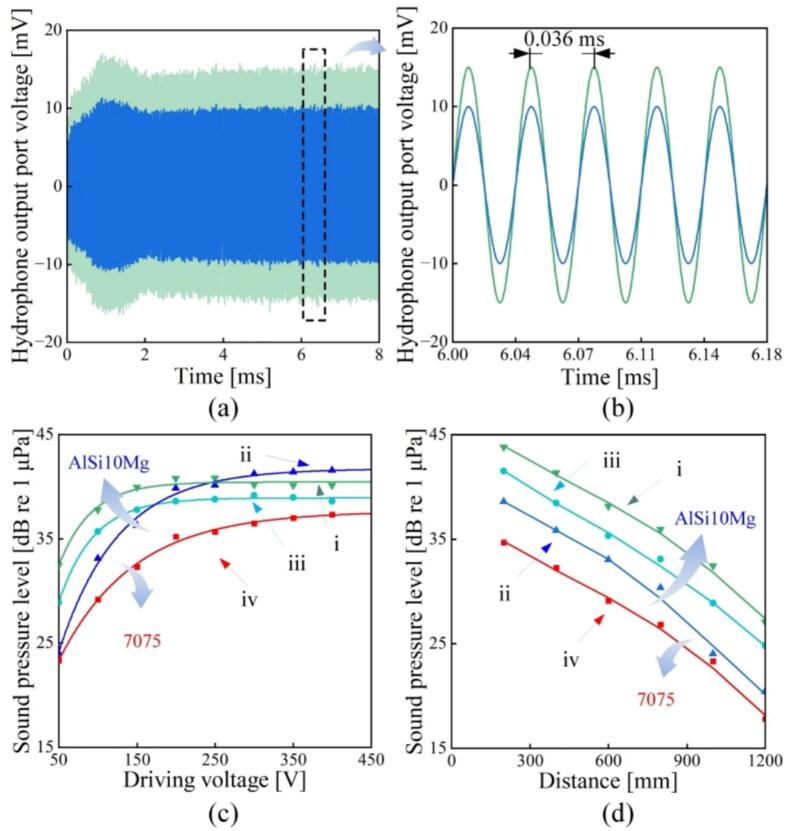
Output voltage of the hydrophone (a) in 0–8 ms and (b) within 5 periods (180 μs). The sound pressure level as functions of (c) the driving voltage and (d) the distance between the transducer and the hydrophone. Note that the distance was kept at 1 m in (c), while the driving voltage was set to 100 V in (d).

Finally, the sound intensities provided with the transducers were measured in a water tank by using a hydrophone (8104, Brüel & Kjær, Copenhagen, Denmark). Fig. 14 shows the experimental setup, where the transducer was immersed into the tank with a rope while the hydrophone was mounted on a bracket with a changeable distance. Fig. 15(a) and (b) show the output voltages of the hydrophone in the periods of 0–8 ms and 6–6.18 ms, respectively. The output voltages of the hydrophone are converted to the sound pressure levels (SPLs) [33]. Fig. 15(c) illustrates the SPLs of the transducers as functions of the driving voltage when the distance is kept at 1 mm. Initially, the SPL exhibits rapid enhancement as the driving voltage increases, and subsequently, the saturation occurs at 200 V for the AlSi10Mg transducers i and iii, and at 300 V for the AlSi10Mg transducer ii and 7075 transducer iv; these results are in consistent with the vibration velocity’s dependence on the driving voltage [see Fig. 12(b)]. Fig. 15(d) plots the variation in SPL against the distance. Clearly, at a certain distance between 200 and 1200 mm, the SPLs provided with the AlSi10Mg transducers exceeded that of the 7075 one; this implies that AlSi10Mg would be a promising material to substitute aluminum alloy for generating intensive ultrasounds in water.

## 5 Conclusions

To examine the feasibility of applying 3D printing metals as the vibrating bodies of ultrasonic transducers, first, we evaluated the mechanical-loss properties of 3D printing metals with varying fabrication parameters. Then, we investigated the vibration properties, temperature rise, and SPL of the AlSi10Mg transducers and conducted the comparison with the 7075 one. The conclusions are as follows.

(1) The damping coefficient of the beam working in the flexural vibration was derived from the distributions of the vibration velocity and the phase; this guarantees the measurement of the damping coefficient’s dependence on the strain and the frequency.
(2) The damping coefficients of AlSi10Mg and 7075 (aluminum alloy) were 1.61 × 10^-4^ and 1.90 × 10^-4^, respectively, at 28 kHz. On the other hand, their damping coefficients were mostly smaller than 316L and 304 (stainless steel alloy) but larger than those of Ti6Al4V (titanite alloy).
(3) The maximum vibration velocity and the SPL of the AlSi10Mg transducers were 1.13 and 1.11 times the values of the 7075 transducer that had the same configuration and operated in the same vibration mode.

These conclusions validate the feasibility to substitute the aluminum alloy (7075) with the 3D printing aluminum (AlSi10Mg) as the vibrating bodies, and pave a new way to fabricate ultrasonic transducers for high-power ultrasonic usage. In the future, we will focus on exploring how other parameters of making 3D printing metals affect the mechanical-loss properties. Besides, we would like to evaluate the suitability of the 3D printing metals in other application areas, e.g., ultrasonic actuators.

## CRediT authorship contribution statement

**Lipeng Wang:** Writing – original draft, Data curation. **Ranxu Zhang:** Software, Data curation. **Jiang Wu:** Writing – review & editing, Project administration, Funding acquisition. **Chengqi Pan:** Software. **Xiaoming Yue:** Visualization, Investigation. **Qiang Zhang:** Validation, Funding acquisition, Formal analysis. **Yibin Li:** Supervision, Conceptualization.

## Declaration of competing interest

The authors declare that they have no known competing financial interests or personal relationships that could have appeared to influence the work reported in this paper.
